# An Insight into the Degradation Processes of the Anti-Hypertensive Drug Furosemide

**DOI:** 10.3390/molecules28010381

**Published:** 2023-01-02

**Authors:** Micaela Giannetti, Viviana Claudia Canale, Laura Micheli, Maurizio Fiori, Claudia Mazzuca, Antonio Palleschi

**Affiliations:** 1 Department of Chemical Science and Technologies, University of Rome “Tor Vergata”, Via della Ricerca Scientifica, 00133 Rome, Italy; 2 Istituto Superiore di Sanità, Department of Food Safety, Nutrition and Veterinary Public Health, Viale Regina Elena, 299, 00161 Rome, Italy

**Keywords:** Furosemide, aging, photo-chemistry, 4-chloro-5-sulfamoylanthranilyc acid, furfuryl alcohol

## Abstract

Furosemide (FUR), an active pharmaceutical ingredient (API) belonging to a group of drugs known as loop diuretics, has widespread use, but, is characterized by a strong instability to light, which causes chemical transformations that could give a yellowing phenomenon and have a significant impact from a health and marketing point of view. Many studies have tried to explain this phenomenon under different experimental conditions, but no detailed explanation of the yellowing phenomenon has been provided. This work, unlike the others, provides an overall view and explanation of the behavior of FUR in relation to the yellowing phenomenon, both in the solution and in solid state, considering several aspects, such as light exposure, presence of oxygen, and moisture effects.

## 1. Introduction

One of the most important problems in the pharmaceutical field, in the production and conservation of final products, is the photo instability of the active principle (API) [1,2,3,4]. Due to photo instability, drugs may indeed form degradation products, lowering the bioavailability of the active principle, but they may also form other highly reactive compounds or light-induced reactions with endogenous substances [4,5,6,7,8].

**Figure 1:**
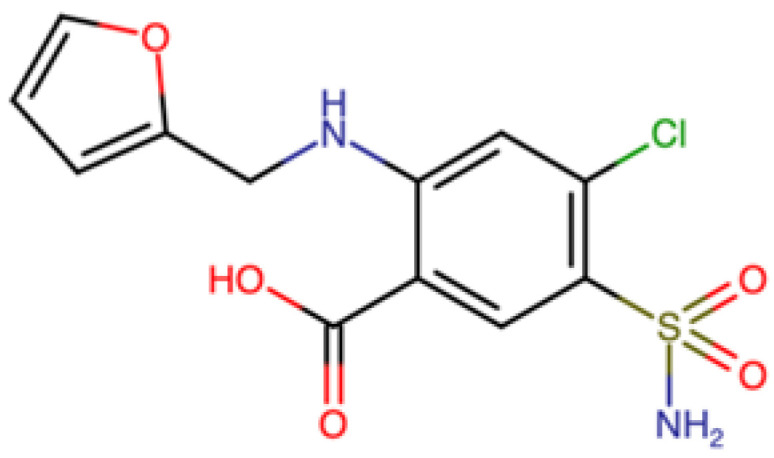
Structure of furosemide.

Furosemide (FUR), or 4-chloro-N-furfuryl-5-sulfamoilanthranilc acid (Figure 1), is an active pharmaceutical ingredient (API) that belongs to a group of drugs known as loop diuretics [9]. This compound causes the excretion of sodium, magnesium, chloride, calcium, and bicarbonate, and it is commonly used for the treatment of oedematous conditions associated with heart, kidney, and liver dysfunction [9]. This API is commonly administered in form of tablets, which also contain several excipients such as lactose, starch, magnesium stearate, and talc. Despite its widespread use, this API is characterized by a strong instability to light, which causes chemical transformations that could cause a yellowing phenomenon [10] and, at the same time, could have a significant impact from a health and marketing point of view.

Many studies have tried to characterize the photodecomposition of FUR under different experimental physical-chemical conditions, such as different pH, light sources, solvents, the presence of oxygen, and temperature [11,12,13,14,15,16]. In this contest, under irradiation, FUR could be subject to dechlorination or decarboxylation with the abstraction of hydrogen and hydroxyl [14,17,18]. Importantly, these reactions are independent of the presence or absence of oxygen. Another important reaction that occurs for exposure to UV light of an aqueous solution of FUR is photo-hydrolysis, which gives rise to a yellow solution containing the degradation products 4-chloro-5-sulfamoylanthranilyc acid (CSA o saluamine) and furfuryl alcohol (FA) (also, in this case the reaction can occur under anaerobic conditions) [19]. These two compounds, in turn, can cause other degradation products, among which are 5-sulphamoylantranilic acid and furfural [12], as well as dimers and polymers through hydrolysis, oxidation or polymerization processes [10], not necessarily involving UV catalysis [5,6] or radical reactions [13].

Anyway, even though the photo-instability of Furosemide has been deeply investigated, there is still no clear explanation about the process conditions that lead to products responsible for yellowing or identification of these colored FUR degradation compounds. Therefore, the aim of the studies reported in this work was to provide an overall view and explanation of the behavior of FUR in relation to the yellowing phenomenon, both in tablet (solid) form and in solution. The choice of considering both forms of residues was to determine the role of water in yellowing. In this context, for example, the presence of excipients such as starch has been investigated. Starch, which is highly hygroscopic, can absorb water and be a reservoir of water for hydrolysis processes [20,21]. To complicate the scenario, other excipients, such as lactose, magnesium stearate, and talc, could react with molecules containing amino groups and cause a browning phenomenon [22,23,24,25,26], as well as cocrystallization with compounds such as urea [27].

Taking this in mind, by using spectroscopic and chromatographic techniques, the occurrence of yellowing phenomena due to FUR degradation was investigated, taking into account several process aspects such as light exposure (both UV and Vis), the absence or presence of oxygen, moisture effects, and the presence of excipients. Therefore, the role of each of these conditions was clarified and reported. 

On the basis of these results, it was possible to establish the optimal process and storage conditions for furosemide-based products to avoid disadvantages. 

## 2. Results and Discussion

### 2.1. Studies in Solid State

The tablets containing furosemide were the target of this research; for this reason, initial studies on solid-state preparation studies, were carried out.

#### Effect of Light

Initially, to investigate the degradation of FUR, several studies have been conducted on the behavior of the API toward light. For this purpose, a set of tablets containing API, or the API and a chosen excipient such as starch and lactose (see below), has been incubated for 1 h in the photoreactor (see Section 3). 

**Figure 2:**
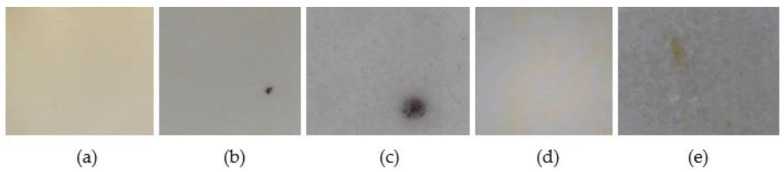
Images taken by a digital microscope of yellowing phenomenon, in tablets containing FUR (**a**), FUR and starch (**b**–**d**), and FUR and lactose (**e**).

As shown in Figure 2, a different kind of yellowing occurs after the exposure, depending on the presence of an excipient. In fact, while the tablet surface of only FUR has shown a uniform colouring, the surfaces of the tablets containing the API and an excipient have shown a spot yellowing.

Furthermore, when analyzing tablets through a digital microscope, black spots with several shapes and intensity were observed on the surfaces of tablets containing FUR or a combination of FUR and an excipient that could be starch or lactose (Figure 2). For comparison, another set of tablets had been stored for eight hours in an aging chamber containing a compact fluorescent lamp, obtaining similar results (data not shown). Importantly, a set of tablets has been put for 24 h in the aging chamber under the inactinic lamp (λ = 550 nm), which, because of its emission wavelength, allowed us to study the effect of visible light. After treatment, no changes in color or FTIR spectra have been observed (Figures S1 and S2). These results demonstrate that the FUR degrades when it is irradiated with UV light. 

To investigate the effect of light, tablets have been characterized through infrared (IR) spectroscopy before and after UV treatment. 

**Figure 3:**
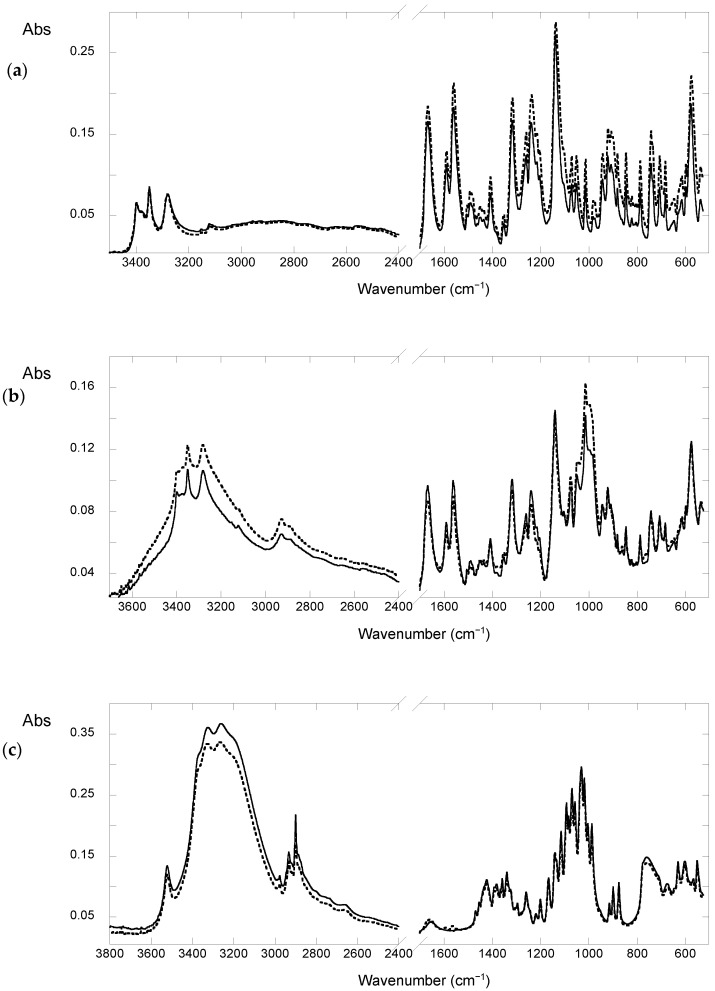

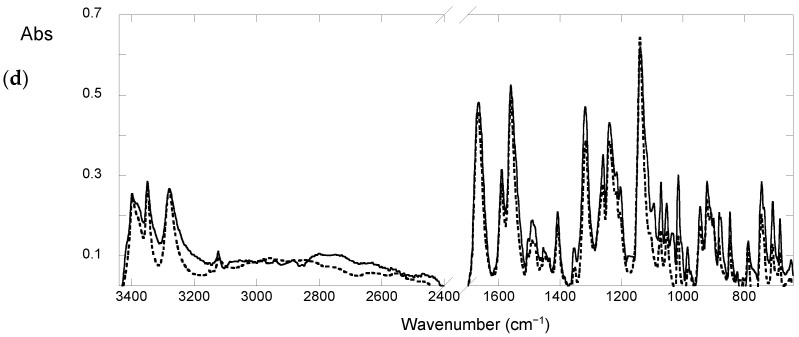
Spectra of (**a**) the yellow (dotted line) and white (straight line) portion of the UV aged furosemide tablet; (**b**) the yellow (dotted line) and white (straight line) portion of the UV aged FUR and starch tablet; (**c**) the white (dotted line) portion of the UV aged FUR and lactose tablet and the spectra of the standard of lactose (straight line); (**d**) the yellow portion (straight line) of the UV aged FUR and lactose tablet and the spectra of the not aged FUR (dotted line).

Regarding the IR spectrum of FUR (Figure 3a), in the region between 3500 and 2800 cm^−1^ there are intense peaks due to the stretching of the NH and OH moieties (3500–3000 cm^−1)^ and less intense peaks due to the asymmetric and symmetric stretches of both the aliphatic and aromatic CH groups (3100–2800 cm^−1^). Furthermore, in the region between 1700 and 1000 cm^−1^, we observed characteristic bands, those attributed to the stretching of the carbonylic groups (at 1668 cm^−1^), the bending of the amine groups (at 1560 cm^−1^ and 1492 cm^−1^), the stretching of the C-O bond (at 1239 cm^−1^), and the stretching of the furan group at 1353 cm^−1^ and 1140 cm^−1^ [28]. It should be noted that in the spectrum of the samples after aging, a broadening of the band at approximately 1138 cm^−1^ attributable to the furosemide dimer, is observable compared to the unaged one. Moreover, as shown in Figure 3b, in the FTIR-ATR spectrum of the yellow part of the tablet containing FUR and starch, there is also a band at 998 cm^−1^ due to the starch itself [29]. This result indicates that yellow spots are characterized by a higher amount of starch, indicating that this excipient plays a role in the formation of the spots. Interestingly, such a process is not photo-induced, as demonstrated by means of UV-Vis aging experiments performed on pure starch that do not give yellowing of this polysaccharide.

The FTIR-ATR spectra of the white parts of the tablets containing FUR and lactose show the typical lactose spectrum, while the spectrum of the yellow spots is equal to the yellow component observed on tablets containing only FUR (Figure 3c,d). From these results, it was possible to conclude that lactose is not responsible for the formation of yellow spots.

**Figure 4:**
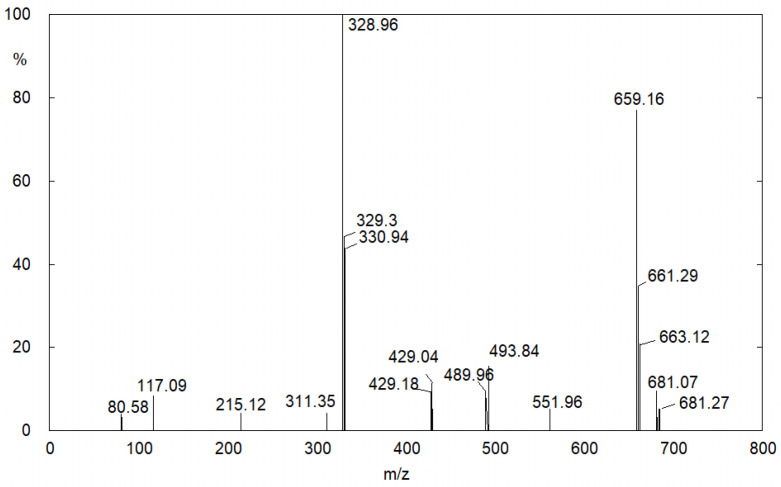
Mass spectrum of the FUR aqueous solution (from tablet) after UV aging.

Because of the presence of yellow spots on tablets containing only the API, it was possible to establish that this photo-induced phenomenon is due to a process that involves the FUR itself. The products that lead to the uniform yellow surface have been identified through UPLC/MS through the analysis of the tablet dissolved in water (see Section 3). The results show that the uniform yellowing of the surface is due to FUR dimers and aggregates of higher molecular weight. From the mass spectrum, reported in Figure 4, it is possible to recognize the FUR peak, *m*/*z* value of 328.96, and the peak at *m*/*z* value of 659.19, double that of the first value, which can be attributable to the FUR dimer. 

### 2.2. Studies in Solution

As reported earlier, FUR can undergo a hydrolytic process under exposure to light. The hydrolytic reaction causes the oxidation of the bond between the secondary amine and the methylene moiety, leading to the formation of CSA and furfuryl alcohol (FA) [19]. Due to this tendency of the API and the effects observed for tablets containing starch or lactose and its hygroscopicity, some studies have been performed directly in aqueous solution. 

To this end, the solution of FUR in water was aged by means of the photoreactor for 1 h. Similar aging has been done for a solution of CSA, which is a product of the FUR hydrolysis. As shown in Figure S3, all solutions turned yellow after the exposure to UV light.

**Figure 5:**
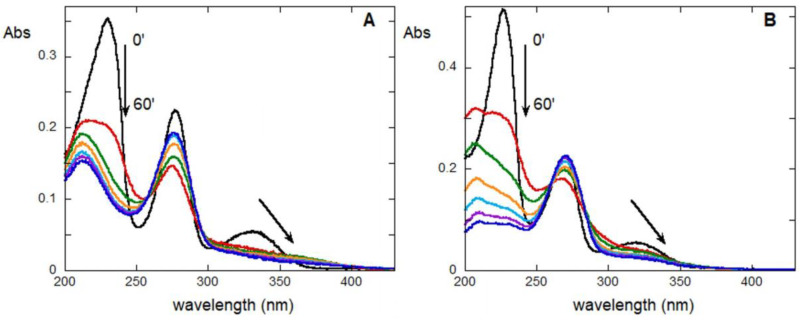
UV-Vis normalized spectra of furosemide (**A**) and CSA (**B**) solutions before (black) and after 10 (red), 20 (green), 30 (orange), 40 (light blue), 50 (violet) and 60 (blue) minutes of UV treatment using the photoreactor.

Using UV-Vis absorption spectroscopy, it was possible to observe similar spectral variations for both ompounds during treatment (Figure 5). Indeed, in both cases, the aging gives rise to a decrease in the molar extinction coefficient of the lowest and highest energy bands, a non-linear variation of those of the band centered at about 272 nm (especially for FUR) and the appearance of an absorption tail between 360 and 400 nm (due to the formation of species responsible to yellowing). Furthermore, spectra show a blue shift of the absorption maximum of the highest energy band as a result of aging and an overall broadening of the bands. 

These results indicate that both FUR and CSA react in a similar way under UV aging. A comparison of the aged spectra of FUR and CSA with that of FA, reported in Figure S4, suggests that the differences could be ascribable to the presence of FA, in the aged or not aged form (see below). This finding supports the idea that in the first stage of aging, FUR hydrolysis occurs, giving rise to CSA and FA that in turn react further. 

**Figure 6:**
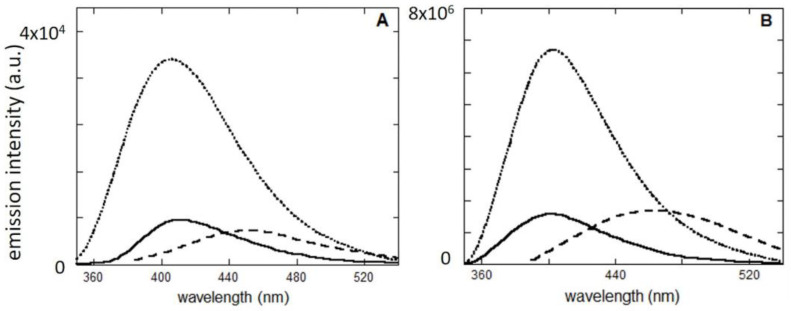
Fluorescence spectra of FUR (**A**) and CSA (**B**) solutions before (dotted line) and after aging with λ_exc_ = 330 nm (dotted and dashed line) and after aging using λ_exc_ = 370 nm (dashed line).

After aging, the occurrence of reactions involving CSA, have been confirmed by fluorescence experiments. As shown in Figure 6, before aging, FUR and CSA show emission spectra centered at 411 and 403 nm, respectively (λ_exc_ = 330 nm). Due to UV treatment, the fluorescence intensity increases in both cases and the two spectra have a maximum at 402 nm. More importantly, after excitation at lower energy wavelength (λ_exc_ = 370 nm), also in both cases, a new emission band appears, which is red shifted with respect to the ones obtained using λ_exc_ = 330 nm (maxima are at 456 nm and 465 nm for Fur and CSA, respectively). These results indicate the presence of new species in the solution, not only in the case of FUR but also for CSA, thus suggesting that degradation does not stop with the formation of CSA. The parallelism between the emission behavior of FUR and CSA strongly suggests that FUR degradation involves CSA production and successive degradation into yellow products. 

In other words, the similarity in the trends observed for both the FUR and CSA is the proof that the hydrolysis reaction participates in the yellowing process and that the cause of the phenomenon is the formation of CSA degradation products. 

**Figure 7:**
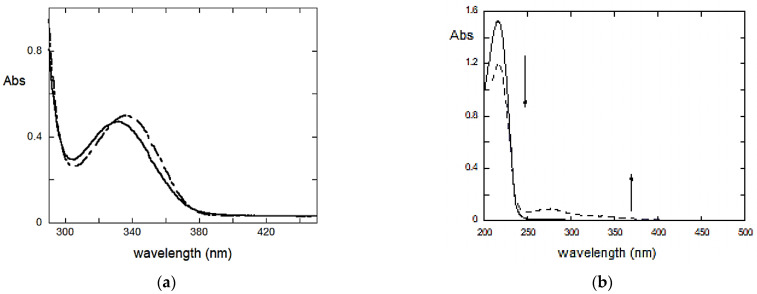
(**a**) Spectra of FUR + ascorbic acid solution before (dotted line) and after (straight line) the aging. (**b**) UV-Vis absorption spectra of a FA solution before (continuous line) and after (dashed line) aging. arrows underline the lowering of the band at 220 nm and the increasing of a band at 370 nm, both due to the aggregation phenomenon.

To validate this hypothesis, similar aging experiments have been performed, adding ascorbic acid (a radical trapper [30]) to the FUR solution. After aging, no color variation of the solution has been observed (Figure S5) and the UV-vis spectrum (Figure 7a) reflects this finding. Indeed very small changes in the spectra are observed, which are compatible with the formation of CSA and FA formation due to FUR hydrolysis, but not with the further production of colored degradation compounds. This proves that a fast yellowing can be attributed to CSA reactions, which occur with a photo-induced and radical process.

Furthermore, the contribution of this to the yellowing of FA must be considered. As reported in the literature [31,32], FA can spontaneously react at room temperature and in the absence of light to form several red- and black-colored polymers and resins such as poly-furfuryl alcohol (PFA) and levulinic esters. Therefore, it is reasonable to attribute this to the dark/reddish spots found in the tablets. 

To confirm this hypothesis, a solution of FA has been aged under the dark for a week in slightly acidic conditions (such as the tablet). During aging, as reported in literature, the solution turned from transparent to reddish/black. 

From the UV-Vis spectrum (Figure 7b), it is easy to see a slight decrease of the band at 210 nm and the formation of a very broad and low intensity peak at 280 nm, with a lower energy tail. These variations with aging are compatible with those observed for the FUR solution, indicating that FA polymerization after FUR hydrolysis is plausible. However, it should be considered that the formation of colored polymers from FA is much slower than that due to CSA.

In contrast, samples aged with an inactinic lamp, that is, under Vis light, can instead be superimposable, thus confirming the ineffectiveness of radiations with λ > 550 nm. In fact, the spectra of the sample before and after the treatment are, in fact, unchanged (Figure S6).

**Figure 8:**
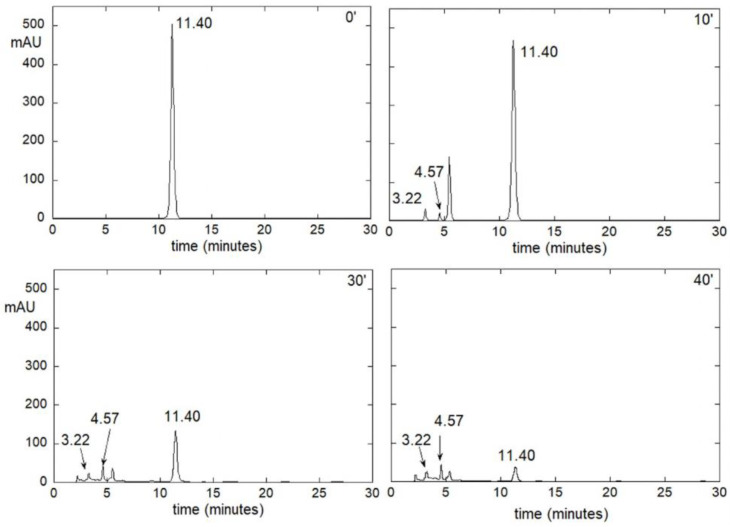
Chromatograms of FUR solution after (from left to right and from upper panel to lower panel) 0, 10, 30, 40 min of UV aging.

**Figure 9:**
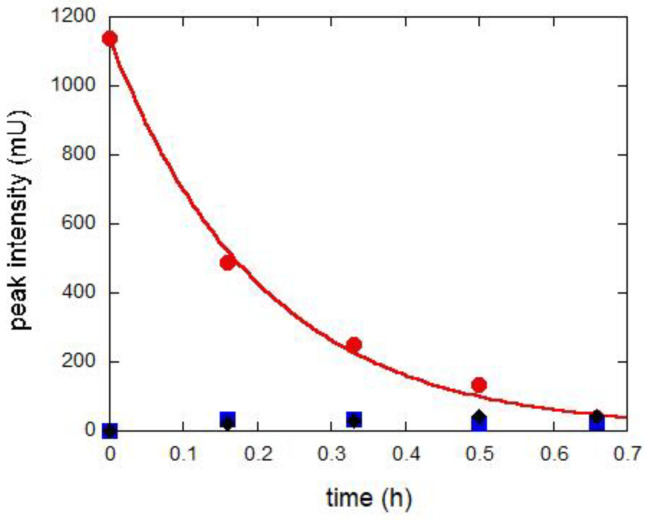
Height variations of the HPLC peaks of FUR (red circles), and CSA (blue squares) and FA (black rhombuses). Continuous line is the exponential fit of FUR data.

**Figure 10:**
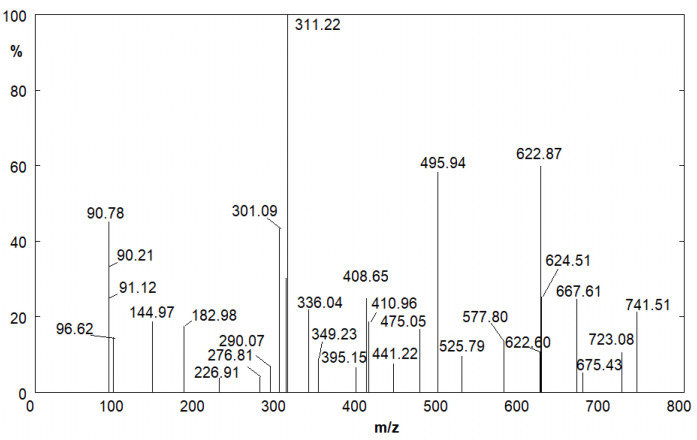
Mass spectrum of the FUR aqueous solution after 40 min of UV aging.

Further confirmation of a FUR degradation mechanism due to UV light has been obtained by HPLC and UPLC/MS analyzes (Figure 8, Figure 9 and Figure 10). 

Concerning the HPLC experiments, a solution of FUR has been exposed to UV aging and analyzed at various growing times (10, 20, 30 and 40 min). As shown in Figure 8, before aging, only the peak at retention time of 11.24 min due to FUR is present. On aging, the intensity of this peak decreases, and other peaks grows; particularly, those with an r. t. of 3.25 and 4.57 min can be attributed, respectively, to the CSA and FA (by means of standards). 

The decrease in FUR peak and the growth of the CSA and FA peaks demonstrate the hydrolytic reaction. It should be noted that FUR has a monoexponential trend (Figure 9), indicating a first-order reaction, with a decay rate of 4.9 ± 0.2 h^−1^, as reported in literature [29,33]. On the contrary, the intensities of the peaks of CSA and FA not only do not show a monoexponential growth trend (Figure S7), but also remain very low onin time. It should be noted, moreover, that with time, the appearance at higher aging times of bands at longer retention times (about 13, 21 and 28 min; see Figure S8), indicating the formation of products characterized by relatively higher molecular weight, occurs. These data suggest the existence of consecutive reactions, in which the first (slower) reaction is the FUR hydrolysis into CSA and FA, and the following ones (faster) are their polymerization.

Polymer formation has been highlighted by the UPLC/MS analysis of the same solutions. The mass spectra show the peak of the FUR at *m*/*z* = 328.96, as in that of tablets (see above), and many other peaks; some of them, on the basis of their *m*/*z* values, can be traced back to the FA polymers and CSA aggregates (Figure 10 and Table 1). 

A scheme of the complex degradation processes of FUR and its derivatives is reported in Figure S9.

### 2.3. Effect of Yellowing on Sticking

**Figure 11:**
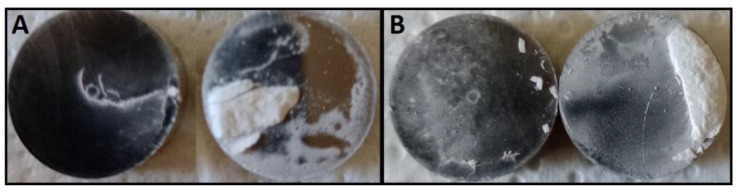
Punches after ejection of tablets containing not aged (**A**) and aged (**B**) furosemide.

Sticking and picking during tablet manufacture has received great interest, as it causes tablet defects, and, consequently, leads to money and time costs. Therefore, knowledge of factors that can affect sticking is therefore useful to provide the appropriate solutions when it arises [34]. Several experiments have been performed to determine the presence of FUR sticking, aged or not. As reported in Figure 11, in both cases, after compression, the ejection steps show defects due to tablet sticking. However, stickiness does not occur if silica (3% of weight), which is a drying agent, is added during tablet formation (data not shown).

## 3. Materials and Methods

### 3.1. Materials

Furosemide, lactose, starch, and CSA were from Merck (Merck KGaA, Darmstadt, Germany); acetonitrile was from Carlo Erba (Carlo Erba Reagenti srl, Cornoredo, Milan, Italy). All reagents were of analytical grade and were used without further purification. Double-distilled Milli-Q water (Millipore, Billerica, MA, USA) was used for the preparation of all solutions.

### 3.2. Tablet Formation

Tablets were prepared by subjecting 200 mg of powder to a pressure of 25 bar through a hydraulic press mod. P400 (Sirio dental srl, FC, Italia). The diameter of the tablets obtained was 1.4 cm. Tablets weighed 200 mg each. In the presence of excipients, the weight ratio for FUR/starch was 1:1 and for FUR/lactose it was 0.6:1 (w:w). Powders were mixed in an Eppendorf by vortexing them. These excipients were chosen because they are present in the most of the recipes of furosemide-based final formulation recipes sold in the market.

### 3.3. Aging of Samples

Photochemical aging has been performed by exposing samples to room temperature for an hour (unless otherwise stated) in a photoreactor (Photochemical Multirays Helios Italquarz Srl, Cambiago, Italy) equipped with UV-Vis lamps (10 × 15 W, 610 ± 10 lux/h) mimicking solar rays (λ_em_ = 365 ± 50 nm). Similar experiments also been performed in a customized irradiation chamber for 24 h equipped with inactinic lamps (Dr. Fisher, 15 W, 7 lm, 14 ± 1 lux/h), mimicking the effect of visible light. The light intensity was measured using a portable luxmeter (Shenzhen Flus Technology Co., Ltd., Shenzhen, China). 

### 3.4. Absorption and Emission UV-Vis Spectroscopy

Solutions of FUR, obtained by solubilizing the API in bidistilled water at different concentrations (0.5 mM, 1 mM and 4.5 mM), have been prepared and characterized before and after the different treatments using UV-Vis absorption spectroscopy using a Cary 100 Scan (Agilent, Billerica, CA, USA). All absorption experiments have been performed using quartz cells with an optical path length of 1 cm or 0.1 cm (Hellma, Italy). Fluorescence UV-Vis spectra on these solutions were obtained using a Fluorolog III instrument. They have been registered using emission and excitation slits of 2.5 nm and a speed scan of 1 nm/s. The excitation wavelengths chosen to register the spectra are 310 nm, 330 nm, and 370 nm. 

### 3.5. FTIR Absorption Spectroscopy

The samples were characterized before and after aging treatments by FTIR spectroscopy, using a Thermo Fisher instrument mod. iS50 (Thermo Fischer Scientific, Madison, WI, USA), equipped with a single reflection ATR diamond cell. For each spectrum, 32 scans were collected with a resolution of 4 cm^−1^. Experiments on colored spots on the tablets have been performed with a SurveyIR™ microscope equipped with a diamond single reflection ATR cell (RedWave Technology Group LLC, Danbury, CT, USA).

### 3.6. Chromatographic Techniques

HPLC analyzes were performed using an Agilent 1100 series Autosampler (G1329A), equipped with four pumps (G1311A) and a 1100 Series Diode-array Detector (Agilent Technologies, CA, USA), with a reverse-phase C18 column (5 μm 250 × 4.6 mm PINNACLE II, RESTEK, USA).

Chromatograms were collected using 70% of water (0.1% formic acid) and 30% acetonitrile isocratic conditions and a flow rate of 1 mL/min. using a detection wavelength λ_1_ = 272 nm. The solutions under analysis are 0.5 mM water/acetonitrile = 1:1 (*v*/*v*) and the injected volume is of 50 μL.

UPLC/MS experiments have been performed through a Waters Acquity UPLC (mod. M1UPA36M, Waters, Milford, MA, USA) combined with a triple quadrupole (mod. Xevo TQ, Waters, Milford, MA, USA) using stepwise gradient solutions. For gradient runs, the following mobile phases were usually used: MILLIQ water with 0.1% formic acid, (solution A) + acetonitrile (solution B) gradient starting at 10% organic phase and increasing to 100% after 10 min for 1.5 min and then returning to 10% for the last 3.5 min. The flow rate was of 0.3 mL/min. MS analysis was performed on the sample with peaks at a retention time of 5.1 min, as it was the most representative of the chromatograms (Figure S10).

## 4. Conclusions

This study sheds light on the key factors that lead to furosemide degradation, which are responsible for the yellowing phenomena that occur in tablets containing this active principle. It is a very important task, as furosemide is increasingly used because of its diuretic properties for the treatment of edematous conditions associated with heart, kidney, and liver dysfunctions; the formation of degradation products is a serious problem from a health and economic point of view. 

In this article, using spectroscopic and chromatographic techniques, we have assessed that the yellowing process comes from two different processes depending on the physical–chemical conditions of the process. In the absence of water, that is., in a tablet, if FUR is exposed to UV light, a uniform yellowing occurs on the tablet surface because of the FUR dimer and a higher-molecular-weight aggregate. On the other hand, in the presence of water, i.e., in an aqueous solution of FUR or in a tablet containing the API and an excipient able to absorb water, such as starch, light exposure induces photo-induced hydrolysis of FUR that leads to the formation of CSA and FA. Then, CSA can lead to the formation of the dimer and aggregates, through a photo-induced radical reaction. The FA, instead, can lead to a polymerization process with the formation of PFA, which results in the dark-colored spots. The latter is not a photo-induced process, but it is an acid-catalyzed process that occurs spontaneously over time. 

Importantly, through these studies, it was possible to determine the process and storage conditions for furosemide-based products, thus minimizing health and economic disadvantages. 

## Figures and Tables

**Table 1:** Assignation of the Mass Spectra peaks reported in Figure 10.

*m*/*z* Value	Compound
96.62	Furfural
226.91	Furfuryl Alcohol trimer
311.22	4-hydroxy-N-furfuryl-5-sulfamoilanthranilc acid
495.94	CSA deprotonated dimer
525.79	Furfuryl Alcohol eptamer
622.84	4-hydroxy-N-furfuryl-5-sulfamoilanthranilc acid dimer
741.51	CSA deprotonated trimer

## Data Availability

Not applicable.

## Supplementary Materials

The following supporting information can be downloaded at: https://www.mdpi.com/article/10.3390/molecules28010381/s1. Additional chromatography and spectroscopic data and data concerning photo aging with inactinic lamp are available. Figure S1: images of furosemide tablets stressed with an inactinic lamp; Figure S2: IR spectrum of the furosemide tablet before and after irradiation with inactinic lamp; Figure S3: Pictures of the aqueous solutions of FUR (a) and CSA (b) after exposure to UV light; Figure S4: UV-Vis spectra of aged solutions of FUR, CSA and FA. Figure S5: Picture of solution of furosemide and ascorbic acid after aging; Figure S6: Picture and UV-Vis spectra of the furosemide solution (4.5 mM) before and after treatment with an inactinic lamp. Figure S7: Chromatogram peaks height variation as a function of time, for CSA (blue) and FA; Figure S8: zoom of chromatogram of 40′ UV aged furosemide solution; Figure S9: Scheme of the degradation process of FUR in the presence and in absence of water; Figure S10: TIC UPLC chromatogram of a surosemide solution aged for 60 min in a photoreactor.
