# Supplementary material for: An Insight into the Degradation Processes of the Anti-Hypertensive Drug Furosemide
Source: Molecules. 2023 Jan 2;28(1):381. doi: 10.3390/molecules28010381 (PMC9823353; doi:10.3390/molecules28010381)
Supplement: Supplementary file 1 [file molecules-28-00381-s001.zip › molecules-2097309-supplementary.pdf]

molecules

MDPI

SUPPLEMENTARY MATERIALS

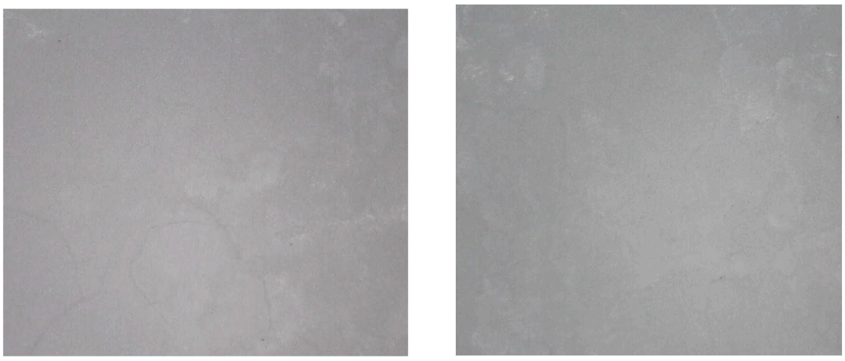

Figure S1. Images of the two facets, obtained by a digital microscope, of a tablet of Furosemide aged by using an inactinic lamp (λ = 550 nm).

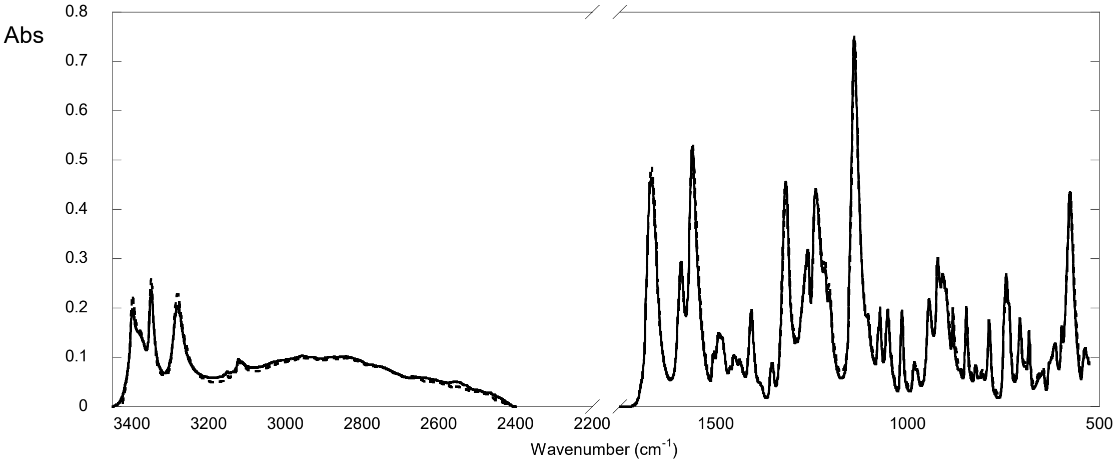

Figure S2. Spectra of Furosemide tablet before (dotted line) and after (straight line) treatment for 8 hours with inactinic lamp (λ = 550 nm).

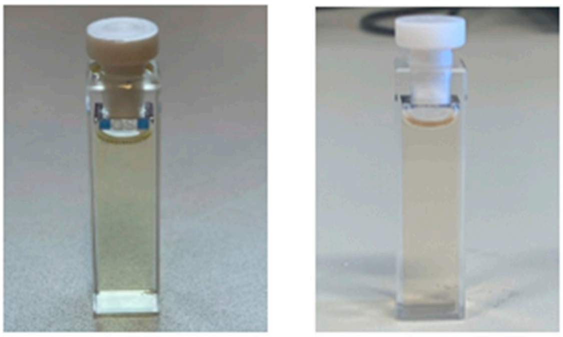

(a) (b)

Figure S3. Pictures of aqueous solutions of FUR **(a)** and CSA **(b)** after exposure to UV light.

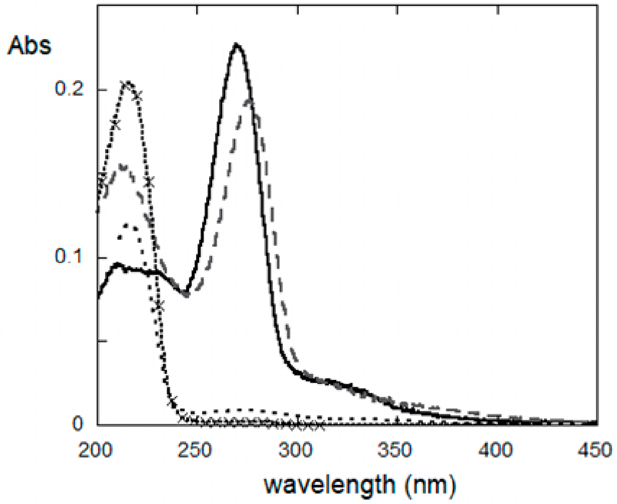

Figure S4. UV-Vis absorption spectra of UV aged FUR (dashed line), CSA (continuous line), not aged FA (dotted line with crosses) and UV aged FA (dotted line).

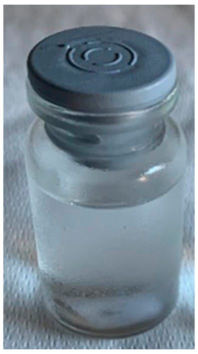

Figure S5. Picture of the solution of Furosemide and ascorbic acid after UV ageing.

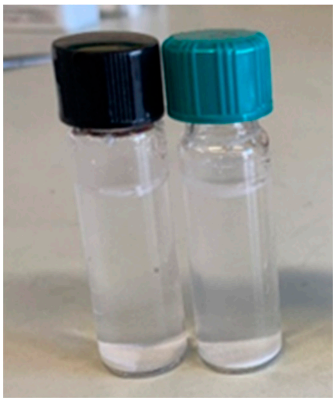

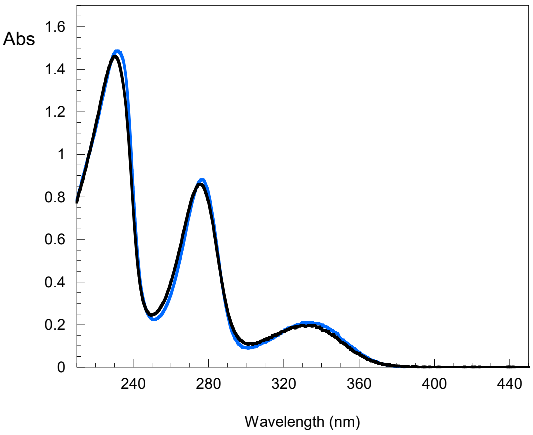

Figure S6. Left: Furosemide solution after treatment with an inactinic lamp. Right: UV-Vis spectra of the Furosemide solution (4.5mM) before (blue) and after (black) the treatment with an inactinic lamp.

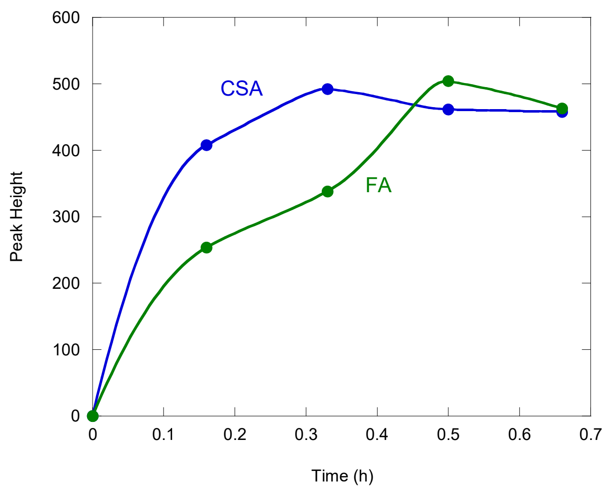

Figure S7. Chromatogram peaks height variation as a function of time, for CSA (blue) and FA (green).

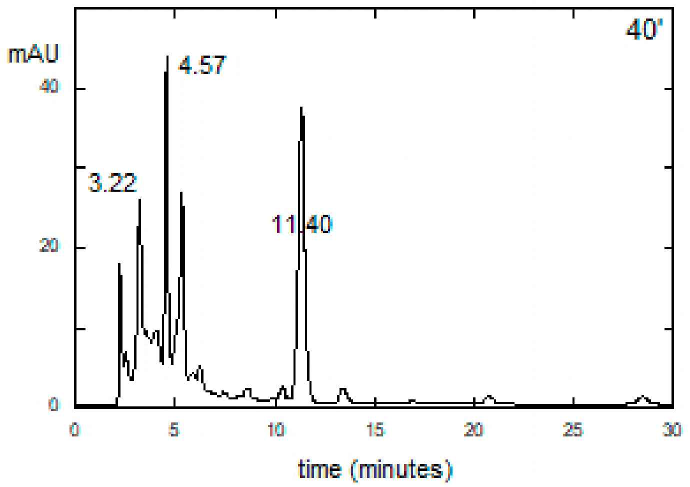

Figure S8. Zoom of the chromatogram of 40 min UV aged Furosemide solution.

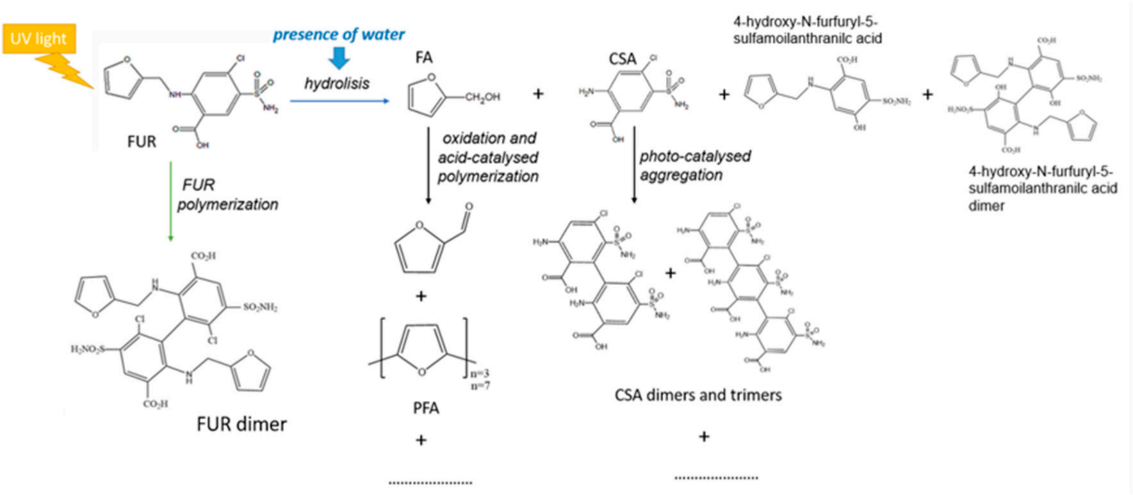

Figure S9. Scheme of degradation process of FUR in presence and absence of water.

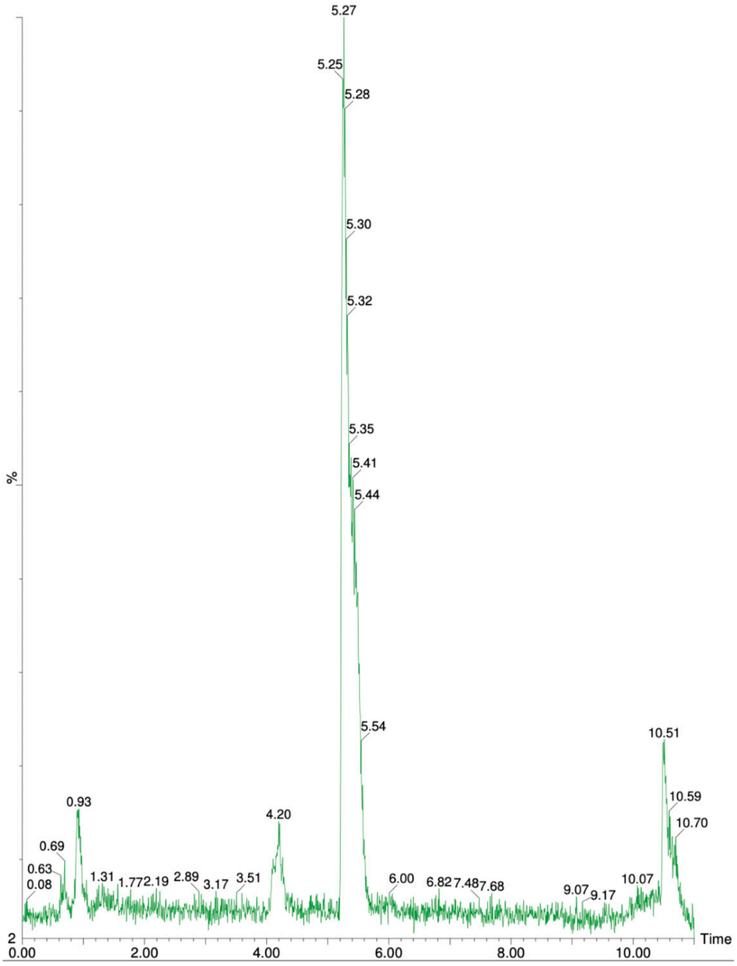

Figure S10: Total Ion Current (TIC) UPLC Chromatogram, taken in Full Scan mode, of a Furosemide's solution treated for 60 minutes in a photoreactor.
